# Microstructure and Corrosion Resistance of AZ91 Magnesium Alloy after Surface Remelting Treatment

**DOI:** 10.3390/ma15248980

**Published:** 2022-12-15

**Authors:** Józef Iwaszko, Monika Strzelecka

**Affiliations:** Department of Materials Engineering, Faculty of Production Engineering and Materials Technology, Czestochowa University of Technology (CUT), 19 Armii Krajowej Ave., 42-200 Czestochowa, Poland

**Keywords:** surface remelting treatment, AZ91 magnesium alloy, GTAW technology, microstructure evolution, corrosion resistance

## Abstract

The effect of surface remelting treatment on the microstructure and corrosion resistance of the AZ91 magnesium alloy was studied. The surface layer was remelted by GTAW (gas tungsten arc welding). An original two-burner system with welding torches operating in a tandem configuration was used, allowing the combination of cleaning the surface from oxides with the remelting process. The studies of the corrosion resistance of the alloy included electrochemical tests and measurements of the rate of hydrogen evolution. The results showed that surface remelting treatment leads to favorable microstructural changes, manifested in strong grain refinement and a more uniform arrangement of the β-Mg_17_Al_12_ phase. The changes in the microstructure caused by remelting and the accompanying fast crystallization contributed to an increase in the corrosion resistance of the remelted samples in comparison to their non-remelted equivalents. The results obtained on the basis of the polarization curves showed three-fold lower values of the corrosion current density in the case of the remelted material than the value of the corrosion current density determined for the starting material. In turn, in the case of measurements of the electrochemical noise and corrosion rate determined by the method of measuring the rate of hydrogen evolution, this value for the remelted alloy was two times lower. The research also showed that GTAW technology is highly effective and can be a valuable alternative to laser techniques. The complete experimental details, obtained results and their analyses are presented in this paper.

## 1. Introduction

Magnesium alloys are modern metallic materials, which, due to their good strength properties and low density (1.74–2.0 g/cm^3^), are increasingly more frequently an alternative for steel and aluminum alloys. This is because the density of those alloys is lower by one-third than that of aluminum alloys and almost 80% lower than the density of steel [1]. In addition, magnesium alloys stand out owing to their good thermal conductivity, good biocompatibility, low heat capacity, excellent machinability and sound damping capabilities and good casting properties, including castability, as well as the possibility of complete recycling [2,3,4]. Despite the unique properties of magnesium alloys, a serious obstacle to their expansion is their low corrosion resistance, which is one of the main causes of the failure of components made of magnesium alloys. Magnesium alloys are susceptible to galvanic corrosion, stress corrosion and fatigue corrosion; however, they have good resistance to intergranular corrosion. The corrosion resistance of magnesium alloys varies with the chemical composition and microstructure. The presence of such elements as iron, copper or nickel, i.e., elements with a higher value of normal potential than magnesium, leads to significant deterioration of the corrosion resistance of the magnesium alloy. These elements play the role of the cathode in a galvanic cell created as a result of the difference in electric potentials. The magnesium then becomes the anode and dissolves. The opposite effect, i.e., improvement in corrosion resistance, is observed when adding zinc or manganese to the magnesium alloy. Corrosion resistance is also dependent on the degree of refinement of the alloy microstructure and on the distribution and morphology of the precipitates. Bearing in mind the importance of magnesium alloys in industry and technology, measures are being taken to improve their corrosion resistance by modifying their chemical composition [5,6], covering the alloy surfaces with protective coatings [7,8,9,10] or applying surface treatments leading to grain refinement [11,12,13,14,15,16].

One of the promising solutions to improve the corrosion resistance of magnesium alloys is the modification of the surface layer microstructure with concentrated heat sources. The use of a laser beam, plasma stream or electric arc allows the concentration of a large amount of heat in a small volume of material, thus inducing rapid crystallization [17,18,19,20,21]. The application of concentrated heat sources in shaping the microstructure and properties of magnesium alloys has recently been investigated by many research teams. For example, Gao et al. [13] applied a 5 kW continuous-wave CO_2_ laser to melt the surface layer of the AZ91HP magnesium alloy. The authors found that in comparison with the alloy in the initial state, the material after laser treatment was characterized by greater grain refinement and redistribution of the β-Mg_17_Al_12_ phase. As a result of changes in the microstructure, the corrosion resistance of the alloy was improved. The microstructure, composition and corrosion resistance of laser surface melted AM60B magnesium were analyzed by Liu et al. [14]. The authors applied a 10 kW continuous CO_2_ laser. In contrast to the alloy in its initial state, the remelted surface layer was characterized by significantly higher corrosion resistance. The authors explain the improvement in corrosion resistance by lower susceptibility to corrosion of the Al-enriched α-Mg matrix and the phase barrier effect, induced by laser remelting. According to the authors, these changes were associated with lowering the corrosion susceptibility of the Al-enriched α-Mg matrix and the barrier effect of the β phase, induced by surface remelting. A ytterbium femtosecond laser was used by Park et al. [22] to melt the surface layer of a biocompatible Mg-Ca-Zn alloy. The authors found that the laser-induced remelting of the secondary phase reduced galvanic corrosion and prevented the occurrence of pitting corrosion, which resulted in a reduction in alloy corrosion. Equally favorable changes in the corrosion resistance of the biocompatible Mg-Zn-Dy alloy were reported by Rakesh et al. [15]. In this case, however, the authors utilized a ytterbium-doped fiber laser. According to the authors, improvement in the corrosion resistance of the laser-melted alloy was mainly due to grain refinement. A nanosecond pulsed fiber laser was employed by Li et al. [16]. Potentiodynamic polarization test results revealed that the corrosion current density values of laser-melted MB26 and AZ80 alloys were reduced about 1.8 and 2.5 times, respectively, compared to the alloys in their initial state. The beneficial effect of surface remelting performed with a CO_2_ laser on the corrosion resistance of AZ91 magnesium alloy was also found by Strzelecka et al. [23].

The analysis of the literature data shows that the surface remelting treatment of magnesium alloys is most often performed employing laser technologies. The short time of the process, the ability to precisely focus the laser beam on a selected surface and the possibility of obtaining very high-temperature gradients in the material—all of this speaks for the use of these technologies. Nevertheless, there are no reports on the use of welding technologies in the surface remelting treatment of magnesium alloys. In the opinion of the authors of this study, GTAW technology may be an equally effective solution. This method, thanks to its undoubted advantages such as the availability and low cost of welding equipment, ease and speed of processing and the low cost of remelting treatment, may in many cases be a competitive solution to the currently dominant laser techniques.

In this study, an attempt was made to improve the corrosion resistance of the AZ91 magnesium alloy by surface remelting treatment. The remelting treatment was carried out by applying GTAW technology, by means of an original two-burner system with welding torches operating in a tandem configuration. This solution was developed and patented by the authors of the article. The changes in the microstructure caused by remelting were also analyzed.

## 2. Materials and Experiment Procedures

The investigated material was the commercially available MgAl9Zn1(AZ91) magnesium alloy obtained by means of the gravity casting method. The chemical composition of the alloy is presented in Table 1.

**Figure 1:**
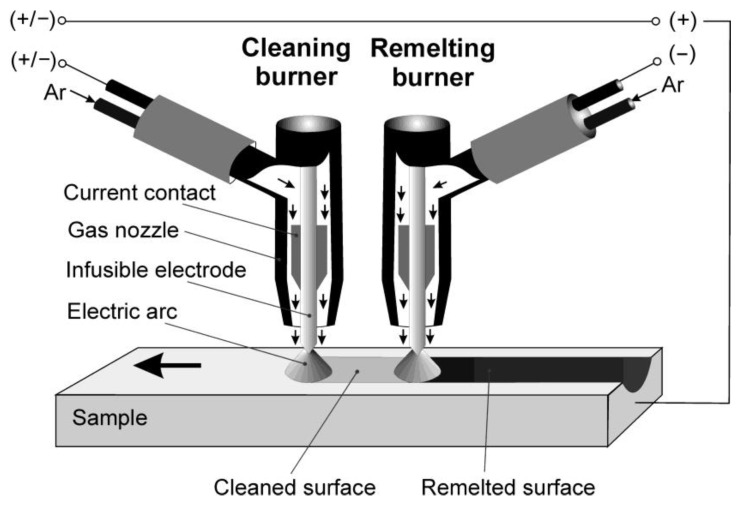
Illustrated model of two-burner tandem system.

Surface remelting treatment was carried out using gas tungsten arc welding technology (GTAW). In this technology, an electric arc glows between an infusible tungsten electrode and the material to be welded. The process takes place in a shield of inert gas flowing from the burner nozzle. The remelting of the surface layer of the AZ91 magnesium alloy using GTAW technology made it necessary to overcome technological problems resulting from the physicochemical properties of magnesium alloys, namely their strong susceptibility to oxidation. Unfortunately, magnesium is a very active metal and reacts with oxygen to form surface oxides. Practice shows that using a protective gas shield during GTAW treatment limits, but does not completely prevent the formation of an oxide layer. The non-conductive oxide layer formed on the surface of the alloy is a barrier to the current, causing deflection of the electric arc and, in extreme cases, even its extinguishing. As a result, the treatment carried out by GTAW technology is accompanied by adverse changes in the geometry of the material surface. To obtain satisfactory effects of remelting, it was necessary to use an unconventional solution. The undertaken work resulted in the development of an original two-burner system working in a tandem configuration, where the task of the first welding torch, powered with an alternate current, was to remove the layer of oxides by cathodic cleaning, and the task of the second, connected to the power supply of a one-directional pulsating current, was to remelt the thus cleaned surface [24,25]. The developed solution has made it possible to combine the process of removing oxides from the surface with the remelting process. An illustrated model of the two-burner tandem system is shown in Figure 1. Detailed data concerning the developed solution are presented in earlier works [24,25].

The surface layer of the AZ91 magnesium alloy was remelted using FALTIG-315 AC/DC and FALTIG-200 DC (OZAS—ESAB Sp. z o.o, Opole, Poland) inverter current sources. The process of remelting and cathodic cleaning of the sample surface was carried out in an argon atmosphere of 99.995% purity. The shielding gas protected not only the electrode and the place to be remelted but also the pool of molten material from atmospheric gases, including oxygen. The presence of oxygen in the atmosphere during the welding of a magnesium alloy is particularly undesirable because it can cause the secondary formation of a refractory oxide layer on the surface of the material, making it difficult or even impossible to carry out the remelting process effectively. The presence of oxygen in the atmosphere could also lead to an increase in the oxygen content in the remelting zone, which would result in the deterioration of its operating parameters.

Remelting was conducted using infusible tungsten electrodes with an addition of thorium dioxide (ThO_2_). Thorium dioxide is added to tungsten electrodes to extend their service life as well as to increase the stability of the arc and facilitate its ignition. Two different tungsten electrodes with diameters of 2.4 and 4 mm were utilized. The electrode diameter was selected on the basis of the welding current used in the remelting treatment.

In the experiment, the constant remelting parameters were the distance between the electrode and the sample surface (L = 2 mm), and the current intensity in the cleaning burner (I = 98 A). Nonetheless, the current intensity in the remelting burner and the traverse speed were changed. The distance between the burners was set at about 25–30 mm and the shielding gas flow at 14 dm^3^/min. Table 2 presents the remelting parameters and the dimensions of the remelted zone.

As can be seen, as the current in the remelting burner increased, the dimensions of the modified zone simultaneously increased, both in terms of the width and depth of material remelting. However, with the current of 150 A, the changes in the surface geometry were so unfavorable that further attempts at remelting with this current were abandoned. In this case, the dimensions of the microstructural changes zone were also not measured either due to a possible measurement error resulting from local material loss in the central part of the band.

Light microscopy examinations were performed by means of a Keyence VHX-7000 light microscope (Keyence Ltd., Osaka, Japan) while scanning electron microscopy investigations were carried out by means of a JEOL JSM-6610LV electron microscope (JEOL Ltd., Tokyo, Japan). Etched metallographic sections cut along planes perpendicular to the remelting direction were observed. The samples were etched with nital.

Phase composition studies were conducted using a Seifert XRD-3003 X-ray diffractometer (Rich. Seifert & Co, Hamburg, Germany) with a cobalt lamp emitting X-ray radiation of the length λ_CoKα1_ = 0.17902 nm. Both the surface-remelted samples and the magnesium alloy in the initial state, which in this case was the reference material, were analyzed. The samples were examined in the range of diffraction angles from 20 to 90 degrees.

EDS studies were performed utilizing an Oxford Instruments EDS microanalyzer (Oxford Instruments Plc., Abingdon, UK).

The investigation of the corrosion resistance of the AZ91 alloy included electrochemical tests (polarization curves, measurements of electrochemical noise) and measurements of the rate of hydrogen evolution. As magnesium exhibits low corrosion resistance in aqueous, diluted chloride solutions, the corrosion resistance was assessed using a corrosive medium in the form of an 0.5 mol dm^−3^ NaCl aqueous solution saturated with Mg(OH)_2_. The saturation of the NaCl solution with magnesium hydroxide ensured that the pH remained unchanged during the corrosion tests.

The results of corrosion tests are highly influenced by the geometric structure of the surface and the specific surface area of the investigated samples resulting from it. The more complex the surface geometry, the greater the contact area with the corrosive agent. For this reason, the samples for the corrosion tests were ground with water-based abrasive papers with a decreasing grain gradation from 1000 to 4000 in order to obtain an identical geometric structure of the surface in all the studied samples. Before testing, the samples were also washed with deionized water and degreased in ethanol.

The electrochemical investigation was conducted employing a CHI760C electrochemical measurement station (CH Instruments, Austin, TX, USA). The samples for the corrosion tests had the shape of cylinders with a diameter of 5 mm. The samples were placed in caps made of polymethyl methacrylate. Additionally, the side walls of the samples were insulated with epoxy resin. The size of the sample working surface exposed to the electrolyte was 0.20 cm^2^. The polarization curves were measured in a 3-electrode system in which the working electrode was made of the AZ91 magnesium alloy, the ancillary electrode was a platinum electrode, and the reference electrode was a saturated calomel electrode (SCE). The adopted research strategy included registration of the polarization curves in the potential range from −2.0 V in relation to SCE up to −1.2 V in relation to SCE. The change in potential proceeded at the rate of 2 mV/s. For each sample, 3 polarization curves were prepared in order to obtain reliable information about the examined material and its corrosion resistance. The electrochemical current noise was measured between two electrodes both made from the alloy in the initial state or from the alloy whose surface was remelted by means of an electric arc (GTAW). The current between the electrodes made of the same material was measured with an electrochemical measuring station CHI760C operating in the electrochemical noise mode. The electrochemical noises were recorded for 24 h; thus, 16,384 measurement points were obtained, each with a sampling frequency of 10 Hz. In order to eliminate the influence of the environmental electromagnetic field on the obtained test results, the measurement system was placed in a Faraday cage. For each data set (σ_I_) the standard deviation of the current and of (σ_E_) the potential was determined. Based on those values (R_n_) the noise resistivity was calculated using the following formula [26]:

(1) $$R_{n}=\frac{\sigma_{E}}{\sigma_{I}}$$

The corrosion rate of the magnesium alloy was also determined on the basis of the rate of hydrogen evolution [27]. As in the case of the other corrosion tests, samples of the magnesium alloy were investigated both in their initial state and after remelting using the GTAW method in order to illustrate the effect of the remelting treatment on the corrosion rate. Only the upper surface of the samples was examined. The side walls and the bases of the samples for the corrosion tests were insulated with a duracrylic resin. Measurements were taken for 48 h and the evolution of hydrogen was recorded every 8 h. In the calculations and analyses, it was assumed that hydrogen is released as a result of the reaction of magnesium with water only.

## 3. Results

### 3.1. Microstructure Characterization

**Figure 2:**
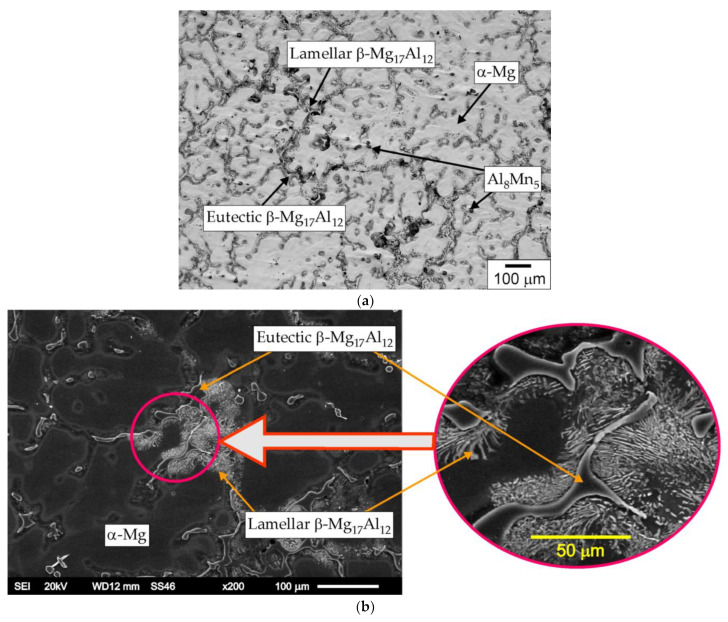
Microstructure of AZ91 alloy, etched cross-section, (**a**) light microscopy, (**b**) SEM.

**Figure 3:**
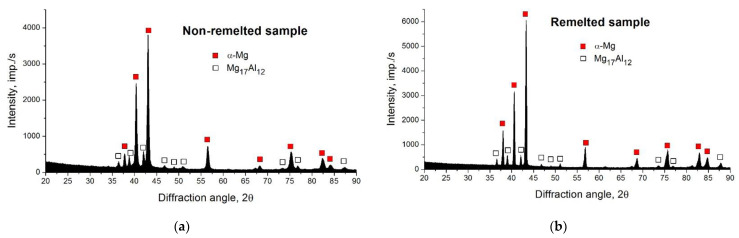
XRD patterns of non-remelted (**a**) and remelted (**b**) samples.

In the first phase of the microstructural studies, the AZ91 magnesium alloy in its initial state was analyzed. An exemplary microstructure of this alloy is shown in Figure 2. The microstructure of the AZ91 alloy consisted mainly of an α-Mg solid solution with an average grain size of about 120 μm. The Jeffries analytical method was used to assess the grain size, and the obtained results were additionally verified utilizing measuring tools included in the software of the Keyence VHX-7000 optical microscope. The dendritic grains of the solid α-Mg solution were separated by a coarse secondary eutectic β phase (Mg_17_Al_12_). The β phase was also a component of the α + β eutectics. The eutectic β phase located at the grain boundaries of the α-Mg phase formed a network throughout the microstructure. Moreover, the presence of small particles of the Al_8_Mn_5_ intermetallic compound characterized by random distribution in the matrix was found. Identification of the phases present in the magnesium alloy was carried out by X-ray phase analysis, with the exception of the intermetallic compound Al_8_Mn_5_, which was identified on the basis of the chemical composition determined by the EDS investigations. X-ray examinations did not reveal the presence of the manganese phase either in the starting material or in the remelted material. This was due to the very low content of this phase, below the X-ray detection threshold. The results of the X-ray phase analysis are shown in Figure 3.

**Figure 4:**
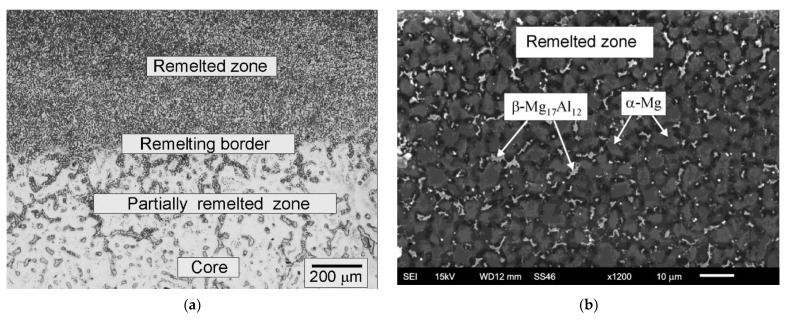
Microstructures of AZ91 magnesium alloy after remelting with current in cleaning burner I = 98 A, and current in remelting burner I = 100 A, (**a**) light microscopy, (**b**) SEM.

The microscopic observations of the remelted samples revealed essential changes in the surface layer microstructure in comparison to the core microstructure (Figure 4). Strong refinement of the microstructure and more even distribution of the individual phases were the main components of the observed microstructural changes. Very fine α phase dendrites dominated in the remelting zone. The dendritic arm spacing in the GTAW remelted surface was in the range of 3–10 μm. In the interdendritic spaces, the presence of β-phase precipitates was also found, which formed a fine network.

The size and nature of the changes found in the surface layer prove that the material was melted and then crystallized rapidly. The occurrence of the rapid crystallization phenomenon is a natural consequence of a high-temperature gradient and a very short time of interaction of the heat source with the examined magnesium alloy.

Apart from the clearly dominant remelting zone, the presence of a partially remelted zone was also found. The formation of the partially remelted zone is primarily a consequence of the low fusion temperature of the eutectics as well as the precipitation of the β phase [28,29,30]. The temperature generated in the material located in the immediate vicinity of the remelted zone exceeds the temperature of the eutectic transformation and the melting point of interdendritic precipitates. The thickness of the partially remelted zone is influenced by a number of factors, namely the thermophysical properties of the magnesium alloy, in particular its heat conductivity and capacity, as well as the employed processing parameters, the size of the element to be remelted and the related heat dissipation rate from the modified zone.

### 3.2. Polarization Curves

**Figure 5:**
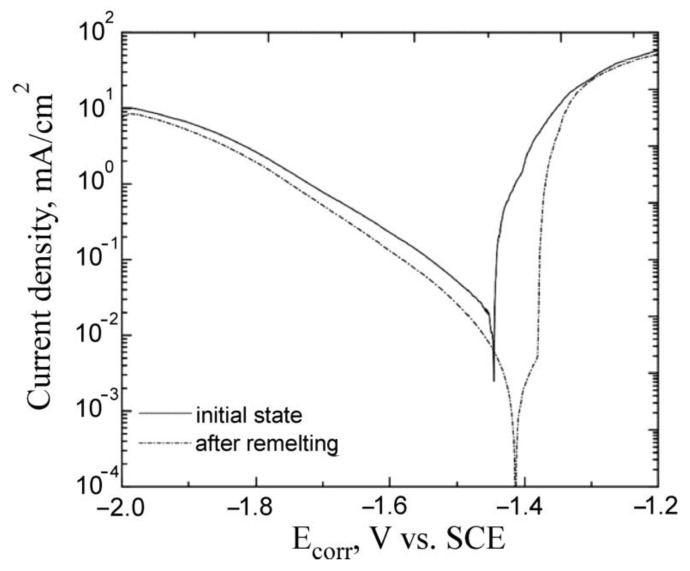
Polarization curves of remelted and non-remelted AZ91 alloy.

The corrosion resistance of the magnesium alloy was assessed on the basis of the polarization curves made by the potentiodynamic method in a NaCl solution. Figure 5 shows the polarization curves received from the AZ91 alloy in the initial state and after remelting using the GTAW method. Based on the recorded polarization curves, the basic corrosion parameters were estimated, namely corrosion potential E_corr_ and corrosion current density j_corr_. To determine the value of the corrosion current density, the method of extrapolation of Tafel straight lines was employed. The obtained potentiodynamic curves clearly indicate that the changes in the microstructure caused by remelting have a significant impact on the corrosion resistance of the magnesium alloy and the course of the corrosion processes in the used environment. In the case of the remelted material, the j_corr_ values were several times lower compared to the material without treatment. The j_corr_ value for the remelted samples was 8.1 µA/cm^2^, whereas for the magnesium alloy in its initial state this value was of the order of 29 µA/cm^2^. According to Faraday’s law, the corrosion rate is proportional to the current density. Thus, the obtained results prove that the corrosion resistance of the remelted AZ91 alloy is higher than that of the starting material. It was also found that the surface remelting treatment causes a shift in the corrosion potential towards positive potential values in relation to the magnesium alloy in its initial state, which proves that the chemical activity of the remelted alloy is reduced in relation to the starting material, and as a result, its corrosion resistance is higher. In analyzing the polarization curves, it can also be seen that remelting causes a decrease in the corrosion current density and a slightly milder increase in the cathode range. Changes in the corrosion current density also entail changes in polarization resistance. Moreover, since the polarization resistance is inversely proportional to the corrosion current density, the lower the current density, the higher the polarization resistance, and the higher its value, the slower the alloy will corrode.

For the remelted samples the E_corr_ value was −1.41 V in relation to SCE, while for the non-remelted ones it was −1.44 V in relation to SCE.

**Figure 6:**
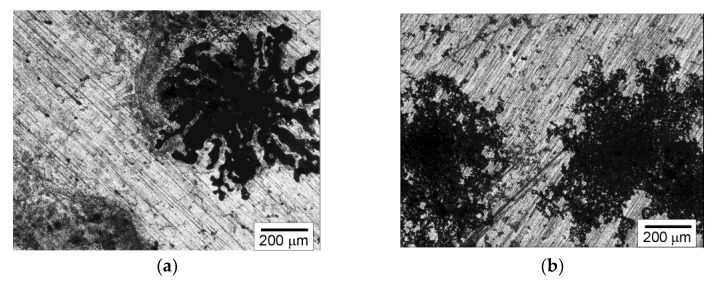
Corrosion centers on non-remelted (**a**) and remelted samples (**b**), light microscope.

After the potentiodynamic corrosion resistance tests, the samples were then subjected to macroscopic analysis to determine the location and size of the corrosion areas on the surface of these samples. The conducted investigations revealed that the AZ91 magnesium alloy was subjected to pitting corrosion. Both in the initial and the remelted samples, the corrosion centers were distributed randomly. There were no places with an above-average number of corrosion centers. However, there were differences in the size and number of corrosion centers in individual samples. It was found that the pits present in the initial samples are clearly greater than in the samples of the material subjected to the remelting treatment. The average diameter of the pits on the surfaces of the remelted samples did not exceed 670 µm, while in the case of the non-remelted samples, the average diameter of the pits was 750 µm. Figure 6 presents examples of pits in the initial (6a) and remelted samples (6b).

### 3.3. Electrochemical Noise

**Figure 7:**
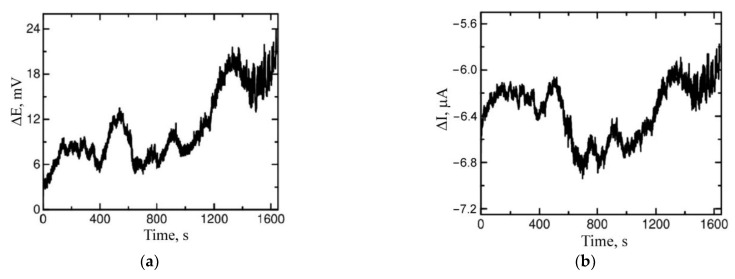
Examplary courses of potential vs. time (**a**) and current noise vs. time (**b**) of remelted AZ91 alloy after 5 h immersion in 0.5 mol/dm^3^ NaCl solution saturated with Mg(OH)_2_.

The next stage of investigations included measurements of electrochemical noise. In an appropriately prepared measurement unit, the courses of the current (I) flowing between two working electrodes made of the same material were recorded during their exposure to a corrosive environment. At the same time, the courses of the potential of the connected (E) electrodes were measured in relation to SCE. In Figure 7, exemplary courses of the potential and current versus time for the samples remelted using the GTAW method are shown. The courses of the potential are presented as the difference between the potential in t time and the potential measured at the beginning of each data set (ΔE).

**Figure 8:**
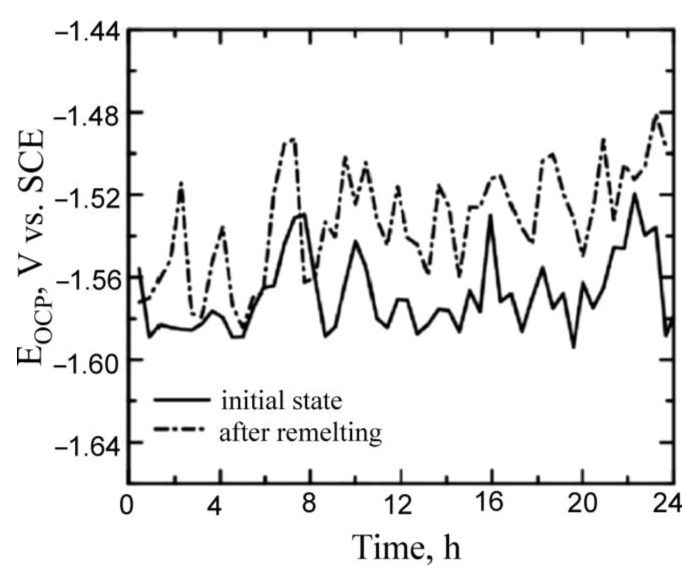
Open circuit potential recorded for remelted and non-remelted AZ91 magnesium alloy.

In Figure 8, the changes in the potential of the electrodes made of the investigated material in relation to SCE, i.e., the changes in corrosion (E_corr_) during 24 h exposure to the corrosive environment are shown. As can be noted, the alloy subjected to surface remelting treatment exhibited a higher corrosion potential than that of the non-remelted alloy. The arithmetic mean of the values of the corrosion potentials during 24 h were −1.53 ± 0.02 V and −1.57 ± 0.02 V in relation to SCE, respectively, for the alloy subjected to remelting and that in its initial state.

**Figure 9:**
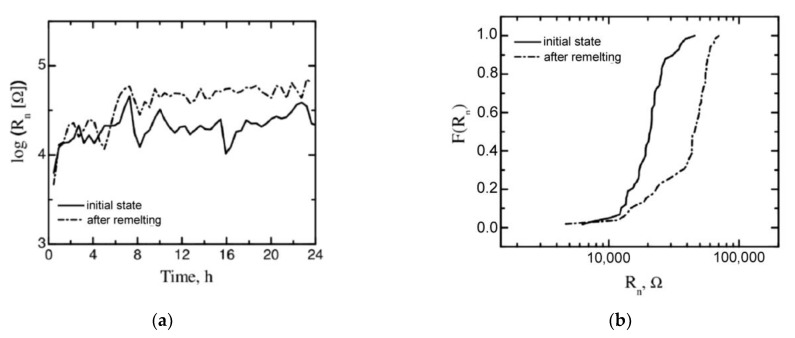
(**a**) Noise resistance obtained for remelted and non-remelted samples; (**b**) cumulative probability plot of R_n_.

Based on the measurements of electrochemical noise, the values of noise resistance R_n_ were determined. Noise resistance is defined as the ratio of the standard deviation of the potential and current noise [26,31]. The changes in noise resistance R_n_ versus time for the remelted samples and their non-remelted equivalents are shown in Figure 9a. Bearing in mind that noise resistance is inversely proportional to the corrosion rate [32], the obtained test results clearly indicate that the magnesium alloy in the initial state is characterized by much lower corrosion resistance than its remelted equivalent. The presented data show that the electrochemical noise resistance value of the samples subjected to surface remelting treatment increased approximately two-fold as compared to the magnesium alloy in its original state, i.e., without remelting.

To increase the clearness of the graph and thereby facilitate the interpretation of the investigation results, a graph of cumulative probability p of noise resistance R_n_ [33,34] was plotted and is presented in Figure 9b. This way of presenting the results allows the distribution of values to be clearly seen, and the plotted values can be easily compared [33]. The presented data explicitly show that the lowest levels of noise resistance were noted for the samples without remelting treatment.

As noise resistance R_n_ can be associated with polarization resistance R_p_, the values of corrosion current density j_corr_ were estimated using the Stern–Geary equation [35]:

(2) $$j_{\mathrm{corr}}=\frac{B}{R_{p}}$$

where:

B—Stern–Geary coefficient (for magnesium 0.064 V),
R_p_—polarization resistance (R_p_ = R_n_).

The average values of the noise resistance recorded during the 24 h measurements were used in the calculations. The arithmetic means of these values were 42.1 ± 16.2 kΩ and 21.6 ± 7.5 kΩ for the alloy subjected to remelting and the alloy in its initial state, respectively.

The most favorable results, and thus the lowest corrosion rates were noted, similar to the polarization curves, for the samples subjected to the remelting process. The j_corr_ value for the remelted samples was 8 µA/cm^2^. An increase, almost two-fold, of the corrosion current density in relation to the remelted material was obtained by the samples without treatment. The j_corr_ value, in this case, was 15 µA/cm^2^.

### 3.4. Hydrogen Evolution Rate Measurements

One of the methods allowing one to determine the material corrosion rate is the method based on measuring the rate of hydrogen evolution [27,36]. This method was employed to evaluate the corrosion rate of the magnesium alloy.

The investigation consisted in measuring the evolved hydrogen volume during contact with the adopted corrosive environment. The volume of evolved hydrogen was converted to the number of moles by applying the Clapeyron equation. In the calculations, corrections for the vapor pressure above the solution and the pressure of the column of liquid in the burette were taken into account. In the calculations, it was assumed that hydrogen is released as a result of the reaction of magnesium with water only. The degradation of magnesium in the aqueous environment takes place by an electrochemical reaction with water, which results in the formation of Mg(OH)_2_ and hydrogen [37,38].

Mg + 2H_2_O → Mg(OH)_2_ + H_2_ (3)

**Figure 10:**
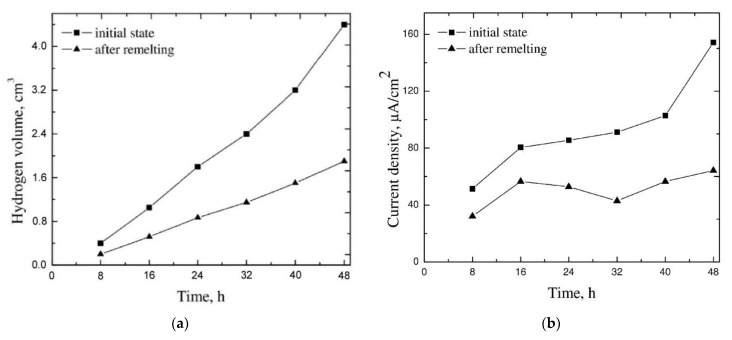
Dependences of hydrogen volume (**a**) and corrosion rates (**b**) on time of immersion in 0.5 mol dm^−3^ NaCl solution for remelted and non-remelted samples.

By using the obtained results, graphs of the volume of evolved hydrogen versus time for the alloy subjected to remelting and its non-remelted equivalent were made. The graphs are displayed in Figure 10a. Owing to the differences in the surface areas of the studied samples, the volume of evolved hydrogen was converted into the rate of corrosion expressed in current density units. The results are presented in Figure 10b. From the presented relation of the current density and time, it can be explicitly seen that the alloy subjected to surface treatment underwent corrosion substantially more slowly than that of the non-remelted alloy. For the alloy subjected to surface treatment, the values of the density of the corrosion current in the course of the experiment were in the range of 32 up to 64 µA/cm^2^, whereas for the non-remelted material they were from 51 up to 154 µA/cm^2^. In both cases, an increase in the corrosion rate with the increase in time of exposure of the investigated materials to the corrosive environment was observed.

## 4. Discussion

This study focuses on research on the effect of surface remelting on the microstructure and corrosion resistance of the AZ91 magnesium alloy. The analysis of the corrosion resistance of the magnesium alloy cannot be carried out in isolation from the analysis of the microstructure of the material and the changes that occur in it as a result of remelting treatment. The microstructure of the material, in the case of an unchanging chemical composition, determines the behavior of the material in a corrosive environment. The use of concentrated heat sources in the remelting treatment of metallic materials causes the microstructure of the material to be formed in specific, non-equilibrium conditions of rapid crystallization. During such processing, an enormous amount of heat is introduced into a small volume of material, which is accompanied by very rapid melting of the material and its equally rapid solidification. The kinetics of this process can be so high that it is even possible to block diffusion processes of nucleation and nucleation growth, which in turn can lead to the amorphization of the material. Nevertheless, such an effect was not observed in the investigated magnesium alloy. Under the conditions of rapid crystallization, phases with a non-equilibrium chemical composition may also be formed, and phase transformations may occur in a different temperature range than during equilibrium cooling. Fast crystallization also affects the grain size in the modified material, causing its significant refinement. Under the conditions of rapid crystallization, a large number of nuclei are formed and the possibility of further growth is effectively blocked. Strong refinement of the microstructure and changes in phase morphology were the main microstructural effects noted in the remelted layer. Remelting, however, did not affect the phase composition of the material, but only the morphology of these phases, which is worth emphasizing. The same main phases, α-Mg and β-Mg_17_Al_12_, were present both in the starting material and in the remelted material, which proves their stability in the conditions accompanying remelting.

The results of the corrosion resistance examinations of the AZ91 alloy before and after the remelting process clearly showed that the surface remelting treatment leads to an increase in the corrosion resistance of the remelted samples in relation to their unmelted counterparts. The reasons for this occurrence include, first of all, strong refinement of the microstructure as well as changes in the morphology and distribution of the β phase noted during the microscopic observations. In the as-received AZ91 alloy the α-Mg grains were very large (the average grain size was about 120 µm), while the β-Mg_17_Al_12_ phase was discontinuously distributed along the grain boundary of the α-Mg. Song et al. [6] report that if the α-Mg solid solution grain size is relatively large and the β phase is discontinuously distributed along the boundaries of the α-Mg phases, the β phase behaves like a galvanic cathode due to the greater potential difference between the α-Mg and β phases.

This situation changed as a result of the remelting treatment. As a consequence of surface remelting, there was a significant reduction in the grain size and uniform distribution of the β-Mg_17_Al_12_ phase in the α-Mg matrix, which contributed to the reduction in the potential difference and thus also the galvanic corrosion. The average grain size of the α-Mg, in this case, was only about 7 μm. With an increase in grain refinement of the α-Mg solid solution; the intervals between the β phase are also smaller and the distribution of the β precipitates is almost continuous. The effect of the β phase on corrosion is then different; namely, this phase acts as a corrosion barrier [6].

The beneficial effect of strong grain refinement on the corrosion resistance of the AZ91 magnesium alloy was found, among others, by Sidhu et al. [39,40]. In turn, Heakal et al. [41] draw attention to the discontinuous distribution of the β phase along the α-Mg grains, which, according to the authors, is one of the factors affecting the low corrosion resistance of the AZ91 magnesium alloy.

It is also worth noting that as a result of the treatment, partial dissolution of the β phase in the matrix takes place; this effect may have an impact on the corrosion resistance of the magnesium alloy. Under certain conditions, the β phase can act as a cathode electrode; therefore, reducing its content may have a positive effect on the corrosion resistance of the AZ91 magnesium alloy. This statement is confirmed by, for example, the results of research conducted by Iranshahi et al. [42] on an electron-beam-treated magnesium alloy. According to the authors, the dissolution of the intermetallic phase during heat treatment provides higher corrosion resistance by reducing microgalvanic corrosion. A more uniform distribution of the β phase and its partial dissolution as a result of remelting leads to a decrease in the ratio of the anode surface area (α phase) to the cathode surface area (β phase), which significantly affects the corrosion resistance of the remelted magnesium alloy [23,43].

Furthermore, Hatakeyama et al. [44] demonstrated that the content of the β phase in the AZ91 magnesium alloy significantly affects the corrosion current density and corrosion resistance in the AZ91 alloy. The presence of the β phase promotes the formation of galvanic cells in the material, as a result of which the α-Mg phase, acting as an anode in the galvanic pair, dissolves quickly [45]. As can be seen, the β phase could play dual roles in the corrosion of α-Mg; it can be either a galvanic cathode or a barrier to corrosion, depending on its size, distribution, and fraction [14,46,47].

In Table 3 and Table 4 the values of the two basic corrosion parameters, i.e., the current density and the corrosion potential for both the remelted and non-remelted samples are listed for comparison purposes. The differences in the values of the corrosion current density determined by various measurement methods for magnesium alloys have been noted by many researchers [27,35,36,48]. The reason for these differences is widely discussed, e.g., in [27]. The differences in the obtained values of the corrosion current density result primarily from differences in the measurement methodology itself. Each of the methods employs different corrosion resistance measurement mechanisms; each of these methods also has its limitations in terms of sensitivity and measurement accuracy and is subject to measurement error. Kirkland et al. [27] point out, for example, that in the case of hydrogen evolution measurement, a potential source of errors or inaccuracies may result from a large number of considerations during the setup and run of the test, which can greatly influence the results and lead to irreproducibility of the tests, but also from the fact that experiments with the flow are generally more difficult to measure. It is also worth noting that the rate of hydrogen release may vary during the test [37]. On the other hand, this method allows analysis of corrosion rate changes occurring during the experiment due to the fact that the measurements are made at multiple time points during the test. Such a possibility is not provided by the polarization method, which is by far the most commonly used method of assessing the corrosion resistance of magnesium alloys. Nonetheless, this method provides information on the instantaneous corrosion rate, and as a result, the measured rate does not reflect the changing nature of the corrosion rate during the test. However, it should be emphasized that the differences found in the current density values do not determine the suitability or unsuitability of a given test method, the more so that each of the employed methods showed that the remelted material has higher corrosion resistance.

Despite the differences in the values of the corrosion current density (Table 3), distinct regularity can be observed. Regardless of the measurement method, the highest values of the corrosion current density, therefore the lowest resistance to corrosion, were observed for the alloy without the treatment. For the remelted alloy, the j_corr_ values were several times lower than those for the non-remelted material. The results obtained in the form of polarization curves showed the highest, a three-fold decrease in the corrosion current density. In turn, for the measurements of the electrochemical noise and the corrosion rate determined by the method of measuring the rate of hydrogen evolution, two-fold lower corrosion current density values were found in the surface-remelted material than those in the initial material.

The corrosion resistance tests were carried out with 3 different methods, therefore, confirming the higher corrosion resistance of the magnesium alloy after surface remelting treatment. This is the key information for assessing the legitimacy of performing such treatment in the case of magnesium alloys. The obtained test results also indicate that each of the employed methods is suitable to assess the corrosion resistance of a magnesium alloy, although the best solution is to measure the corrosion rate using several different techniques and then compare the obtained results. Thanks to this, it is possible to obtain more reliable results and the risk of incorrect assessment of the corrosion resistance of the material is reduced. The intention of the authors of this paper was to employ three different methods of corrosion resistance testing in order to confront these methods with each other, but above all to minimize the possibility of drawing incorrect conclusions regarding the corrosion resistance of the magnesium alloy.

The values of the corrosion potentials for the remelted and the non-remelted samples are presented in Table 4. The compiled results obtained by measuring the polarization curves and electrochemical noise demonstrate that the surface remelting treatment leads to a shift in the corrosion potential (E_corr_) towards positive potential values in comparison to the non-remelted material.

## 5. Conclusions

Based on the research and analysis of the obtained results, the following conclusions can be drawn:

1. Surface remelting treatment leads to favorable changes in the microstructure of the material. Strong refinement of the microstructure and more even distribution of the individual phases are observed.
2. As a result of surface remelting treatment, β-phase precipitates are partially dissolved in the magnesium matrix.
3. Changes in the microstructure and phase morphology in the remelted samples are a consequence of rapid crystallization caused by a high-temperature gradient and rapid cooling of the material.
4. The corrosion resistance of the surface-remelted AZ91 magnesium alloy is significantly improved, mainly owing to the strong grain refinement and redistribution of the β-Mg_17_Al_12_ phase.
5. Remelting the surface layer of the AZ91 magnesium alloy using gas tungsten arc welding technology may be an alternative solution to laser techniques, and because of its competitive price, ease of use and availability of welding equipment, it seems to be a particularly interesting solution.

## Figures and Tables

**Table 1:** Chemical composition of AZ91 magnesium alloy.

Alloy	Element Content, wt%						
MgAl9Zn1 (AZ91)	Al	Zn	Mn	Si	Fe	Cu	Mg
	8.5	0.7	0.32	0.01	0.001	0.001	rest

**Table 2:** GTAW remelting parameters.

No	Current Intensity in Remelting Burner I, A	Scanning Speed V, mm/s	Electrode Diameter d, mm	Bandwidth W_p_, mm	Depth of Remelting G_p_, mm
1	80	5	2.4	6.70 ± 0.05	1.45 ± 0.05
2	100	5	2.4	6.81 ± 0.05	2.13 ± 0.05
3	110	5	2.4	8.50 ± 0.05	2.68 ± 0.05
4	120	5	4	7.09 ± 0.05	2.73 ± 0.05
5	150	5	4	unfavorable changes in surface geometry	
6	150	8.5	4		

**Table 3:** Comparative list of corrosion current density values determined by different measurement methods.

Type of Sample	Measurement Method of Corrosion Current Density j_corr_		
	Polarization curves	Electrochemical noises (mean values)	Hydrogen evolution rate measurements (values after 48 h)
Initial state	29 µA/cm^2^	15 µA/cm^2^	154 µA/cm^2^
After remelting	8.1 µA/cm^2^	8 µA/cm^2^	64 µA/cm^2^

**Table 4:** Comparative list of corrosion potential values determined by different measurement methods.

Type of Sample	Measurement Method of Corrosion Potential vs. SCE, E_corr_	
	Polarization curves	Electrochemical noises (mean values)
Initial state	−1.44 V	−1.57 V
After remelting	−1.41 V	−1.53 V

## Data Availability

The data that support the findings of this study are available from the corresponding author, J. Iwaszko, upon reasonable request.

## References

[1] Ramaiyan S., Chandran R., Santhanam S.K.V. (2017). Effect of cooling conditions on mechanical and microstructural behaviours of friction stir processed AZ31BMg alloy. Mod. Mech. Eng..

[2] Catar R., Altun H. (2019). Investigation of stress corrosion cracking behaviour of Mg-Al-Zn alloys in different pH environments by SSRT method. Open Chem..

[3] Zemková M., Minárik P., Jablonská E., Veselý J., Bohlen J., Kubásek J., Lipov J., Ruml T., Havlas V., Král R. (2022). Concurrence of high corrosion resistance and strength with excellent ductility in ultrafine-grained Mg-3Y alloy. Materials.

[4] Kulekci M.K. (2008). Magnesium and its alloys applications in automotive industry. Int. J. Adv. Manuf. Technol..

[5] Zhang T., Meng G., Shao Y., Cui Z., Wang F. (2011). Corrosion of hot extrusion AZ91 magnesium alloy. Part II: Effect of rare earth element neodymium (Nd) on the corrosion behavior of extruded alloy. Corros. Sci..

[6] Song Y.L., Liu Y.H., Yu S.R., Zhu X.Y., Wang S.H. (2007). Effect of neodymium on microstructure and corrosion resistance of AZ91 magnesium alloy. J. Mater. Sci..

[7] Carcel B., Sampedro J., Ruescas A., Toneu X. (2011). Corrosion and wear resistance improvement of magnesium alloys by laser cladding with Al-Si. Phys. Procedia.

[8] Wasserbauer J., Buchtík M., Tkacz J., Fintová S., Minda J., Doskocil L. (2020). Improvement of AZ91 alloy corrosion properties by duplex Ni-P coating deposition. Materials.

[9] Hajiali Fini M., Amadeh A. (2012). Corrosion resistance of AZ91 magnesium alloy with pulse electrodeposited Ni-SiC nanocomposite coating. J. Nano Electron. Phys..

[10] Fritzsch K., Zenker R., Buchwalder A. (2015). Improved surface properties of AZ31 and AZ91 Mg alloys due to electron beam liquid phase surface treatment. Mater. Today: Proc..

[11] Abbasi M., Bagheri B., Dadaei M., Omidvar H.R., Rezaei M. (2015). The effect of FSP on mechanical, tribological, and corrosion behavior of composite layer developed on magnesium AZ91 alloy surface. Int. J. Adv. Manuf. Technol..

[12] Dobrzański L.A., Tański T., Malara S. (2011). Effect of the heat and surface laser treatment on the corrosion degradation of the Mg-Al alloys. Mater. Eng. Mater. Inžinierstvo.

[13] Gao Y., Wang C., Yao M., Liu H. (2007). Corrosion behavior of laser melted AZ91HP magnesium alloy. Mater. Corros..

[14] Liu C., Liang J., Zhou J., Wang L., Li Q. (2015). Effect of laser surface melting on microstructure and corrosion characteristics of AM60B magnesium alloy. Appl. Surf. Sci..

[15] Rakesh K.R., Bontha S., Ramesh M.R., Das M., Balla V.K. (2019). Laser surface melting of Mg-Zn-Dy alloy for better wettability and corrosion resistance for biodegradable implant applications. Appl. Surf. Sci..

[16] Li Y., Arthanari S., Guan Y. (2019). Influence of laser surface melting on the properties of MB26 and AZ80 magnesium alloys. Surf. Coat. Technol..

[17] Du J.-Y., Li F.-Y., Li Y.-L., Wang L.-M., Lu H.-Y., Ran X.-J., Zhang X.-Y. (2019). Influences of plasma arc remelting on microstructure and service performance of Cr_3_C_2_-NiCr/NiCrAl composite coating. Surf. Coat. Technol..

[18] Iwaszko J., Strzelecka M. (2016). Effect of cw-CO_2_ laser surface treatment on structure and properties of AZ91 magnesium alloy. Opt. Laser. Eng..

[19] Dong T., Zheng X., Li G., Wang H., Liu M., Zhou X., Li Y. (2018). Effect of TIG remelting on microstructure, interface and wear resistance of Fe-based coating. J. Eng. Mater. Technol..

[20] Li Y., Dong T., Li G., Wang H., Fu B., Zheng X., Zhou X. (2018). Microstructure and mechanical property of Ni-based thick coating remelted by gas tungsten arc. Vacuum.

[21] Szafarska M., Iwaszko J. (2012). Laser remelting teratment of plasma-sprayed Cr_2_O_3_ oxide coatings. Arch. Metall. Mater..

[22] Park J., Han H.-S., Park J., Seo H., Edwards J., Kim Y.-C. (2018). Corrosion behavior of biodegradable Mg-based alloys via femtosecond laser surface melting. Appl. Surf. Sci..

[23] Strzelecka M., Iwaszko J., Malik M.A. (2016). Corrosion Resistance of AZ91 Magnesium Alloy after Laser Remelting Treatment. J. Wuhan Univ. Technol. Mater. Sci. Ed..

[24] Iwaszko J., Strzelecka M., Kudła K. (2017). Surface modification of AZ91 magnesium alloy using GTAW technology. Bull. Pol. Acad. Sci. Tech. Sci..

[25] Szafarska M., Iwaszko J., Kudła K., Łegowik I. (2013). Utilisation of high-energy heat sources in magnesium alloy surface layer treatment. Arch. Metall. Mater..

[26] Gouveia-Caridade C., Pereira M.I.S., Brett C.M.A. (2004). Electrochemical noise and impedance study of aluminium in weakly acid chloride solution. Electrochim. Acta.

[27] Kirkland N.T., Birbilis N., Staiger M.P. (2012). Assessing the corrosion of biodegradable magnesium implants: A critical review of current methodologies and their limitations. Acta Biomater..

[28] Zhu T., Chen Z.W., Gao W. (2008). Microstructure formation in partially melted zone during gas tungsten arc welding of AZ91 Mg cast sheet. Mater. Charact..

[29] Shen J., You G., Long S., Pan F. (2008). Abnormal macropore formation during double-sided gas tungsten arc welding of magnesium AZ91D alloy. Mater. Charact..

[30] Zhu T., Chen Z.W., Gao W. (2006). Incipient melting in partially melted zone during arc welding of AZ91D magnesium alloy. Mater. Sci. Eng. A.

[31] Smulko J., Darowicki K., Zieliński A. (2002). Detection of random transients caused by pitting corrosion. Electrochim. Acta.

[32] Zhang T., Shao Y., Meng G., Wang F. (2007). Electrochemical noise analysis of the AZ91D magnesium alloy in alkaline chloride solution. Electrochim. Acta.

[33] Sanchez-Amaya J.M., Cottis R.A., Botana F.J. (2005). Shot noise and statistical parameters for the estimation of corrosion mechanisms. Corros. Sci..

[34] Zhang T., Liu X., Shao Y., Meng G., Wang F. (2008). Electrochemical noise analysis on the pit corrosion susceptibility of Mg–10Gd–2Y–0.5 Zr, AZ91D alloy and pure magnesium using stochastic model. Corros. Sci..

[35] Pardo A., Feliu S., Merino M.C., Arrabal R., Matykina E. (2010). Electrochemical estimation of the corrosion rate of magnesium/aluminum alloys. Int. J. Corros..

[36] Williams G., Birbilis N., McMurray H.N. (2013). The source of hydrogen evolved from a magnesium anode. Electrochem. Commun..

[37] Cesarz-Andraczke K., Nowosielski R., Sakiewicz P., Babilas R. (2018). Metody badania odporności korozyjnej stopów magnezu do zastosowań w implantologii medycznej. LAB Lab. Apar. Bad..

[38] Zhen Z., Ting-fei X., Yu-feng Z. (2013). A review on in vitro corrosion performance test of biodegradable metallic materials. Trans. Nonferrous Met. Soc. China..

[39] Sidhu H.S., Singh B., Kumar P. (2021). Effect of cryogenic treatment on corrosion behavior of friction stir processed magnesium alloy AZ91. Mater. Today Proc..

[40] Sidhu H.S., Singh B., Kumar P. (2021). To study the corrosion behavior of friction stir processed magnesium alloy AZ91. Mater. Today Proc..

[41] Heakal F.E.-T., Bakry A.M. (2019). Electrochemical characterization of certain Mg-based alloys in artificial perspiration biofluid for consumer and industrial applications. J. Mater. Eng. Perform..

[42] Iranshahi F., Nasiri M.B., Warchomicka F.G., Sommitsch C. (2022). Investigation of the degradation rate of electron beam processed and friction stir processed biocompatible ZKX50 magnesium alloy. J. Magnes. Alloys..

[43] Ramalingam V.V., Ramasamy P., Kovukkal M.D., Myilsamy G. (2020). Research and development in magnesium alloys for industrial and biomedical applications: A review. Met. Mater. Int..

[44] Hatakeyama M., Shimono K., Iwashima D., Saikawa S., Sunada S. (2017). The role of β(Al_12_Mg_17_) phase on corrosion behavior of the AZ91 alloy in NaCl aqueous solution. Arch. Metall. Mater..

[45] Saxena A., Singh Raman R.K., Bobby Kannan M. (2008). Laser assisted surface modification of AZ91 alloy: Microstructural and electrochemical study. Trans. Indian Inst. Met..

[46] Song G.L., Atrens A., Dargusch M. (1999). Influence of microstructure on the corrosion of die cast AZ91D. Corros. Sci..

[47] Zhang S., Jiang J., Zou X., Liu N., Wang H., Yang L., Zhou H., Liang C. (2022). Progress of laser surface treatment on magnesium alloy. Front. Chem..

[48] Shi Z., Cao F., Song G.L., Atrens A. (2014). Low apparent valence of Mg during corrosion. Corros. Sci..

